# Gastric adenocarcinoma in the excluded stomach 18 years after bariatric surgery: a case report

**DOI:** 10.1093/jscr/rjac448

**Published:** 2022-09-20

**Authors:** Rodrigo Piltcher-da-Silva, Vivian Laís Sasaki, Luiz Francisco Cravo Bettini, Gabriel Jasinski, Beatriz Carolina Schuta Bodanese, Guilherme Vieceli Rhoden, Tiago Zibetti dos Passos, Guilherme Francisco Gomes, Quézia Tizo Santos, Yan Sacha Hass Aguilera, João Augusto Nocera Paulin, Júlio Cezar Uili Coelho

**Affiliations:** General and Digestive Surgery Service, Hospital Nossa Senhora das Graças, Curitiba, Brazil; General and Digestive Surgery Service, Hospital Nossa Senhora das Graças, Curitiba, Brazil; General and Digestive Surgery Service, Hospital Nossa Senhora das Graças, Curitiba, Brazil; General and Digestive Surgery Service, Hospital Nossa Senhora das Graças, Curitiba, Brazil; General and Digestive Surgery Service, Hospital Nossa Senhora das Graças, Curitiba, Brazil; General and Digestive Surgery Service, Hospital Nossa Senhora das Graças, Curitiba, Brazil; Digestive Endoscopy Service, Hospital Nossa Senhora das Graças, Curitiba, Brazil; Digestive Endoscopy Service, Hospital Nossa Senhora das Graças, Curitiba, Brazil; Radiology Service, Hospital Nossa Senhora das Graças, Curitiba, Brazil; General and Digestive Surgery Service, Hospital Nossa Senhora das Graças, Curitiba, Brazil; General and Digestive Surgery Service, Hospital Nossa Senhora das Graças, Curitiba, Brazil; General and Digestive Surgery Service, Hospital Nossa Senhora das Graças, Curitiba, Brazil

**Keywords:** gastric cancer, bariatric surgery, gastric surgery, Roux-en-Y gastric bypass, vein thrombosis

## Abstract

Gastric cancer (GC) ranks fourth in overall cancer mortality. Bariatric surgical procedures, especially the gastric bypass surgery (GBS), raise a concern about the risk of GC in the excluded stomach (ES). Diagnosis of GC in the ES is challenging due to anatomical changes and impossibility of endoscopic access to the ES. There are few reports of GC after GBS, and it occurs more in the gastric stump than in the ES. We report a case of a 54-year-old female with GC in the ES 18 years after GBS. The increasing number of GBS and the aggressiveness of the GC show how relevant this case is to emphasize the need to consider this diagnosis in patients who develop upper abdominal symptoms, anemia or weight loss.

## INTRODUCTION

Gastric cancer (GC) ranks fifth in incidence and fourth in overall cancer mortality worldwide [1]. It is estimated that 256 000 bariatric surgeries were performed in the USA in 2019 and nearly 60% of these operations were sleeve gastrectomy (SG) [2]. Despite SG’s upsurge worldwide as a trend for treating metabolic syndrome and morbid obesity, gastric bypass surgery (GBS) is still the technique of choice in Brazil [3]. One important concern about GBS is the limitation of endoscopic access to the excluded stomach (ES).

Chronic *Helicobacter pylori* infection is the main cause of noncardia GC; its global prevalence is 50%, reaching 80% in resource-limited nations [1, 4]. However, <5% of these individuals will develop GC. Genetic and environmental factors are also important predisposing factors to GC [1]. The protective and risk factors of noncardia GC are presented in Table 1 [1, 2, 5].

**Table 1:** Risk and protective factors for noncardia GC

Risk factors	Protective factors
*H. Pylori* infection [1, 5]	Less salt and salted food intake [1, 5]
Alcohol consumption [1, 5]	Intake of fruit and vegetables [1, 5]
Tobacco smoking [1, 5]	Vitamin A intake [5]
Salt and salt-preserved foods [1, 5]	
Blood group A [5]	
Processed meat [1, 5]	
Grilled or smoked meat and fish [1, 5]	
Older age [5]	
Low socioeconomic status [5]	
Previous gastric surgery (Billroth I and II) [5]	
Familial predisposition [5]	
Pernicious anemia [5]	
Familial adenomatous polyposis [5]	
Lynch syndrome [5]	
Cowden syndrome [5]	
Juvenile polyposis [5]	
Li–Fraumeni syndrome [5]	
Mutyh-associated adenomatous polyposis [5]	
Peutz–Jeghers syndrome [5]	
Hereditary diffuse gastric cancer [5]	

In the obese individual, an increased incidence of cancer is observed [6]. In the gastric stump, up to a 5-fold increase in the risk of GC has been described after 15–25 years of surgery [7]. However, there are few reports of GC in the ES, occurring 5–22 years after surgery [6, 8]. The diagnosis is challenging since there is no access to perform upper gastrointestinal endoscopy (UGE) in the ES and the symptoms are nonspecific [9, 10]. The most important preventive measure is the systematic preoperative risk assessment, with a detailed evaluation of familial history, patients’ conditions and UGE for assessment of the presence of *H. pylori* [7]. We present a case of a 54-year-old woman with GC located in the ES 18 years after she had been subjected to a GBS.

## PRESENTATION OF CASE

A 54-year-old woman presented with a 30-day history of upper abdominal pain. Although she was taken omeprazole since the onset of symptoms, the pain worsened in the last week. There were no complaints of weight loss or changes in bowel habits. At evaluation, she was stable and had pain in the epigastrium. The medical record has shown that she underwent GBS 18 years ago with no complications and maintains regular follow-up with bariatric surgery team. There was no relevant family history.

**Figure 1:**
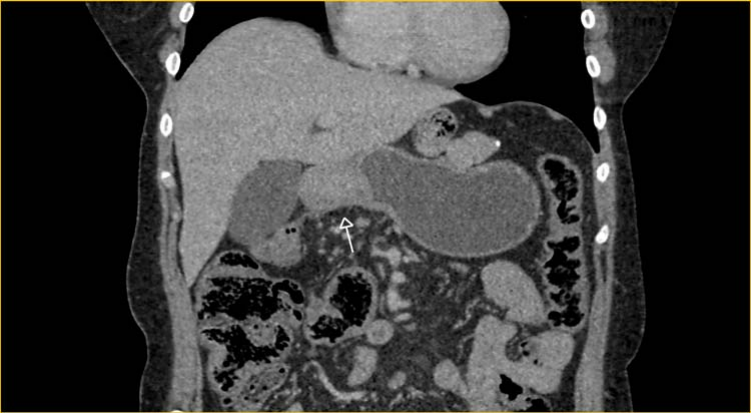
Portal phase coronal section image on CT showing excluded stomach distention with parietal thickening of the pylorus and antrum (arrow).

**Figure 2:**
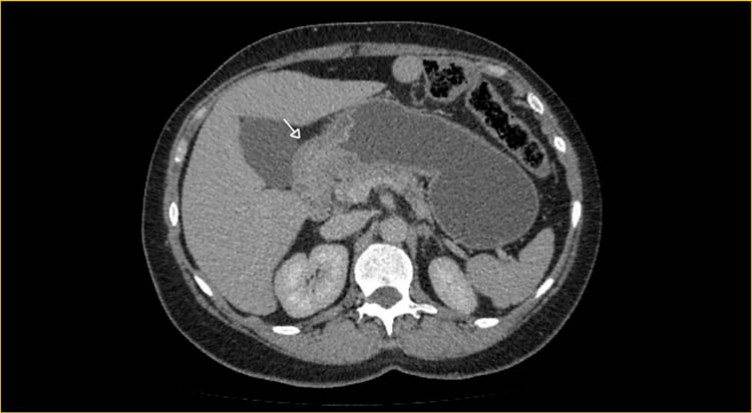
Portal phase transversal section image on CT showing excluded stomach distention with parietal thickening of the pylorus and antrum (arrow).

**Figure 3:**
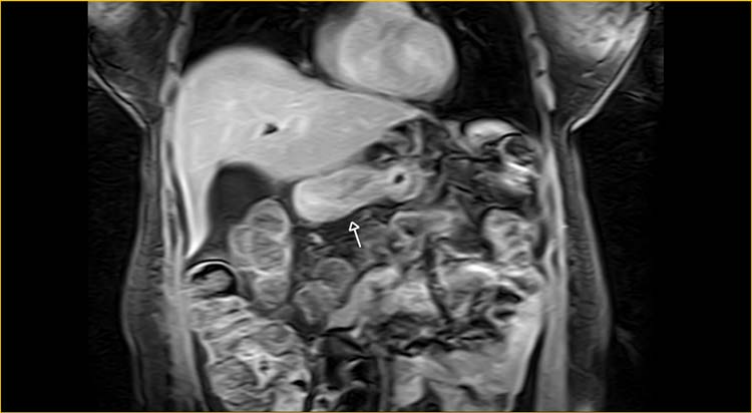
Coronal section on contrast magnetic resonance showing excluded stomach distention with parietal thickening of the pylorus and antrum (arrow).

**Figure 4:**
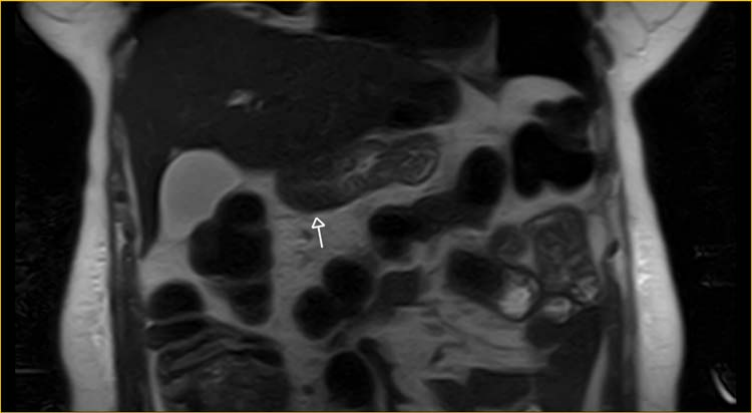
Transversal section on T2-weighted magnetic resonance showing excluded stomach distention with parietal thickening of the pylorus and antrum (arrow).

Laboratory examination showed anemia. An excluded stomach distention and parietal thickening of the pylorus and antrum were identified during abdominal computed tomography (CT) and magnetic resonance imaging (Figs 1–4). Thoracic CT was normal.

The patient underwent combined laparoscopy with intraoperative endoscopy, which revealed an expansive lesion in the antrum. There was no metastasis. The biopsies confirm poorly differentiated diffuse type adenocarcinoma. The patient underwent uneventful ES gastrectomy with D2 lymphadenectomy. The anatomopathological exam of the stomach confirmed the diagnosis of poorly differentiated gastric adenocarcinoma, diffuse type, with serosal invasion and angiolymphatic and perineural invasion. All 27 lymph nodes removed were showed no metastasis (pT3pN0pM0).

She remained in the intensive care unit for 2 days. On the second (POD), diet was started. Abdominal drain was removed on the seventh POD and the patient was discharged. She was readmitted to the hospital on the 10th POD due to eventration. She underwent abdominal wall closure and was discharged 23 days later.

## DISCUSSION

Obesity has reached pandemic levels in the last decades. Since 1975, the incidence of obesity has increased 8% worldwide [11]. It is expected that the diagnostics of GC in post-GBP patients will expand [11].

Obesity has a systemic carcinogenic effect, resulting in an increased risk of developing cancer in various organs, such as liver, pancreas and stomach [7]. Although the underlying mechanisms are not completely elucidated, various theories have been proposed, such as pro-inflammatory effect due to metabolic syndrome, chronic hyperinsulinemia exposing preneoplastic cells to tumorigenic effects and development of insulin resistance [9, 12]. The weight loss obtained after BS also seems to be responsible for the reduced risk of cancer in these patients [13].

When the tumor develops in the ES, where there is no access by conventional endoscopy, the diagnosis is challenging [9, 10]. Early symptoms are nonspecific, such as anemia, nausea, abdominal discomfort and distention [7]. CT may be an option for monitoring formerly obese patients, but is neither a sensitive exam to detect early lesions nor cost/effective [9, 10].

A meta-analysis showed only 17 articles between the years 2002 and 2013 on gastric cancer after BS, 6 of 17 (35.29%) were adenocarcinoma after GBP and 5 of 6 (83.33%) were in the ES [6]. Even with the relative protection promoted by GBP, by diverting ES exposure to exogenous carcinogens, the development of GC in this case may be due to pancreatobiliary reflux, a well-known cause of metaplasia [6, 7].

Endoscopic findings in ES were accessed by using a double-balloon enteroscopy in 40 patients, with success in 35 patients (87.5%) of these: ES normal (25.7%), erythematous gastritis (28.6%), erosive gastritis (28.6%), atrophic gastritis (17.1%) and intestinal metaplasia (5.7%) [8]. Biliary reflux was found in 24 patients (68.6%), and 7 patients (20%) tested positive for *H. pylori*. According to BS protocol, the bacterium was eradicated during preoperative assessment [8].

As a consequence of the late diagnosis of GC in ES, the cancer stage tends to be advanced. Oncologic gastrectomy is the main treatment [9]. So, for obese individuals with intestinal metaplasia in the distal stomach or multiple predisposing factors for GC on preoperative evaluation, subtotal gastrectomy should be considered [7].

The data concerning GC in the ES are scarce. Tinoco *et al*. found only 1 in 3047 patients who underwent BS between 1999 and 2014 [14]. Long-term follow-up is recommended; the diagnosis of GC in the ES should be considered in bariatric patients with new onset of epigastric pain, gastric plenitude, abdominal distension, anemia or weight loss [10].

## CONCLUSION

The growing number of BS, as well as the risk of GC, should cause concern among surgeons and raise awareness of this prospective event. Epidemiologic studies are needed to deeply investigate this association.

Most of the patients who undergo GBP surgery are young, and they will have pancreatobiliary reflux for decades without adequate assessment. We suggest that adequate monitoring of this population needs to be evaluated.

## CONFLICT OF INTEREST STATEMENT

The authors declare that they have no conflict of interest and that the ethical principles were followed.

## FUNDING

This study did not receive any specific grant from funding agencies in the public, commercial or non-profit sectors.

## ETHICS APPROVAL

This study complies with institutional/national ethical standards. There is no need for evaluation by National Research Ethics Commission to short report.
